# Characterization of antibiotic determinants and heavy metal resistance genes in Escherichia coli from pigs in Catalonia

**DOI:** 10.1099/mgen.0.001371

**Published:** 2025-03-25

**Authors:** Biel Garcias, William Monteith, Anna Vidal, Laia Aguirre, Ben Pascoe, Carolin M. Kobras, Matthew D. Hitchings, Samuel K. Sheppard, Marga Martin, Laila Darwich

**Affiliations:** 1 Department Sanitat i Anatomia Animals, Veterinary Faculty, Universitat Autònoma de Barcelona (UAB), Cerdanyola del Vallès, CP 08193, Spain; 2 Ineos Oxford Institute for Antimicrobial Research, Department of Biology, University of Oxford, South Parks Road, Oxford, OX1 3RE, UK; 3 Sir William Dunn School of Pathology, University of Oxford, South Parks Road, Oxford, OX1 3RE, UK; 4 Swansea University Medical School, Singleton Park, Swansea SA2 8PP, UK

**Keywords:** antimicrobial resistance, *Escherichia coli*, heavy metal tolerance, plasmids, swine farming

## Abstract

More antibiotics are administered to livestock animals than to treat human infections. Industrialization, large animal densities and early weaning mean pigs are exposed to more antibiotics than any other livestock animal. Consequently, antimicrobial resistance (AMR) is common among commensal and pathogenic bacteria. Heavy metals (HMs) are also often used as feed additives for growth promotion and infection prevention alongside antimicrobials, and increased exposure to copper, zinc and cadmium can further encourage AMR through co-selection. In this study, we sequenced an archived collection of 112 *Escherichia coli* isolates from pigs in Catalonia using short- and long-read sequencing methods to detect AMR and HM tolerance genes. The most common AMR genes were *mdfA* (84.8%), *aph(3″)-Ib* (52.7%), *bla*_TEM-1B_ (45.6%) and *aph(6)-Id* (45.6%). Genes relevant to public health, such as the extended-spectrum *β*-lactamases (15.4%), *bla*_CTX-M_ type or *bla*_SHV_, or mobile colistin resistance (*mcr*) genes (13.4%), such as *mcr-1*, were also found. HM tolerance genes were present in almost every genome but were rarely located in plasmids, and, in most cases, AMR and HM tolerance genes were not located on the same plasmids. Of the genes predicted to increase tolerance to HMs, only those with activity to mercury were co-located on plasmids alongside other AMR determinants. However, mercury is rarely used in pig farming and does not support a scenario where AMR and HM genes are co-selected. Finally, we identified the exclusive association between *mcr-4* and ColE10 plasmid, which may help target interventions to curtail its spread among pig *Escherichia coli*.

**Impact Statement**

The global use of antimicrobials and heavy metals (HMs) in farmed livestock as feed additives to promote growth and prevent infections has contributed to the spread of resistance among commensal and pathogenic bacteria of these animals. We investigated antimicrobial resistance (AMR) and HM tolerance in 112 *Escherichia coli* isolates from pigs in Catalonia using short- and long-read sequencing techniques to identify AMR and HM tolerance genes. While HM tolerance genes were widespread, they rarely co-located with AMR genes on the same genetic elements. AMR genes were mainly found in plasmids, while HM tolerance genes were principally located in chromosomes. The *mcr-4* gene was exclusively associated with the ColE10 plasmid, suggesting the possibility of targeted interventions to curb its spread in pig *Escherichia coli* populations.

## Data Summary

Assembled genomes and supplementary material are available from FigShare (https://doi.org/10.6084/m9.figshare.24541513.v2). Short and long raw reads were deposited in the GenBank database in the National Center for Biotechnology Information (NCBI) under the BioProject PRJNA1040819.

## Introduction

Since penicillin was first discovered in 1928, antimicrobials have transformed our society and economy, saving countless human and animal lives. Antibiotics have been used routinely in farm animal production since the 1950s to treat, control and prevent infectious diseases and to increase productivity. However, the incongruous use of antimicrobials in human and animal medicine over the last few decades has promoted the emergence and spread of antimicrobial resistance (AMR), which has since become one of the greatest global health challenges of our time [1]. Due to recent industrialization, high-density packing numbers and earlier weaning, swine are exposed to more antibiotics than any other livestock animal [2], and consequently, AMR is widespread in the industry [3,6]. Many antibiotics categorized as critically important antimicrobials (CIAs) for human medicine according to the World Health Organization (WHO CIA list), such as enrofloxacin or ceftriaxone, are commonly used in animals [7], and subsequent resistance can directly affect human well-being. Several AMR genes that provide resistance to last-resort antibiotics reserved for the most severe infections in humans have been detected in swine [8,9].

Plasmid-mediated genes that confer colistin resistance in commensal *Escherichia coli* have been isolated from pigs, pork products and humans in China [10]. Deemed too toxic for human use, colistin is reemerging as a last-line antibiotic in clinical medicine for use against multidrug-resistant (MDR) bacteria that are resistant to conventional antimicrobial therapies [11]. Colistin use has previously been widespread in agriculture and the swine production industry, including for treating *Escherichia coli* infections in post-weaning cases of diarrhoea. This has promoted the emergence of colistin-resistant isolates, either through point mutations in the chromosome [12] or plasmid-mediated colistin resistance genes. Mobile colistin resistance (*mcr*) genes, such as *mcr-1*, pose a significant public health risk as they can be easily transferred between bacteria, leading to onward transmission from livestock to humans [10]. Livestock swine are a key reservoir of *mcr* genes [13,14], among other AMR genes of public health relevance, including extended-spectrum *β*-lactamases (ESBLs) and plasmid-mediated quinolone resistance genes [15,18].

An epidemiological link has been shown between antimicrobial consumption in animals and increased AMR in humans [19]. However, identifying a direct link between AMR resistance isolates found in livestock and those that affect humans is not always easy as there are often limitations in sampling and timeframes [15]. Studies of *Klebsiella pneumoniae* [16], *Escherichia coli* [17] and *Enterococcus faecium* [18] from both livestock and human sources have had limited success, demonstrating a clear One Health transmission network [20]. Furthermore, commensal bacteria can act as a genetic reservoir from which disease-associated clones emerge [20,22] as evidenced by the emergence of pathogenic lineages of *Streptococcus suis* in European and Asian pig stocks [20,21]. This can extend to AMR genes, where the greater diversity in commensal strains can be accessed by pathogenic lineages [22,23]. Many AMR genes have evolved from these environments, and novel resistance genes such as *mcr-1* are often first identified in isolates from livestock (mainly swine) or aquaculture industries [24,25], emphasizing the requirement for a One Health approach to AMR surveillance [26].

In addition to antimicrobials, heavy metals (HMs) such as zinc oxide or copper sulphate have been used extensively in the swine industry. Benefits for piglets after weaning include increased growth rate, immunostimulation, antioxidant and an anti-inflammatory effect that helps improve intestinal integrity [27,30]. Researchers have previously demonstrated an AMR increase after HM use [31,32], and metagenome studies have shown evidence of AMR and HM co-selection in swine [29,33, 34], and co-location of both compounds in the same plasmid is plausible.

*Escherichia coli* is commonly used as a sentinel species to assess AMR [30,32, 35], and whole-genome sequencing of *Escherichia coli* strains provides an effective surveillance tool to monitor AMR in swine [3,36,38]. Alongside clonal transmission of resistance determinants [39], plasmids are expected to play a pivotal role in AMR dissemination. In this study, we characterize the genomes of 112 *Escherichia coli* isolates from farmed pigs in Spain, particularly in Catalonia, a region known for its prominent role in Spanish pig production. Our objective was to identify known determinants of AMR and genes involved in HM tolerance to pinpoint patterns of co-selection and assess their extra-chromosomal mobility.

## Methods

### Bacterial genome collection

The Veterinary Laboratory for the Diagnosis of Infectious Diseases (VLDID) of the Universitat Autònoma de Barcelona (Spain) has a biobank of frozen bacterial strains isolated from porcine clinical cases, sampled between 1999 and 2020. A representative subset of *Escherichia coli* isolates was selected from different studies conducted in pig farms with a record of neonatal or post-weaning diarrhoea [14,40]. In total, 112 isolates (96 from diarrhoeic piglets and 16 from healthy pen mates) were sampled from swine farms, and pure isolate cultures were stored at −80 °C in Microbank® beads (Pro-Lab Diagnostics, Cheshire, UK). The selected isolates were thawed and cultured on McConkey agar under aerobic conditions for 24 h at 37 °C as previously described [40].

### DNA extraction, short-read sequencing and archiving

DNA was extracted using the QIAMP DNA Mini Kit (QIAGEN, Crawley, UK), as per the manufacturer’s instructions. Nucleic acid content was quantified using a NanoDrop spectrophotometer before normalization and sequencing. Libraries were prepared using the Nextera XT Library Preparation Kit, and high-throughput sequencing was performed using an Illumina MiSeq sequencer (Illumina, Cambridge, UK). Libraries were sequenced using a 2×300 bp paired-end version 3 reagent kit (Illumina). Short-read paired-end data were filtered and trimmed, and adapted sequences were removed using Trimmomatic (default settings, version 0.39) [41] and assembled using SPAdes (default settings, version 3.7) [42]. FASTQC [43] was used to assess quality, and all assembled genomes with an estimated average sequencing depth greater than 25× and N50 above 20 000 bp were uploaded to the Public Databases for Molecular Typing and Microbial Genome Diversity (PubMLST) (https://pubmlst.org/) [44] and given a unique identification number (PubMLSTid; Table S1, available in the online Supplementary Material).

### Phylogenetic analysis and phylogroup and sequence type identification

Multiple sequence alignments were made from concatenated sequences using the blast algorithm to identify orthologues of genes present in the reference genome following a gene-by-gene alignment [45,46], with *Escherichia coli* K12 strain (accession number: NZ_CP010444.1) as a reference, using MAFFT (version 7.525, default parameters) [47]. The core genome was defined as genes shared by 90% or more of the isolates, and the subsequent alignment was used to construct a maximum-likelihood phylogeny using RAxML version 8 [48] with the General Time Reversible-GAMMA substitution model [49]. Interactive phylogenies were visualized using Microreact (https://microreact.org/project/iXryyFQq1p9Z6G5uV9Fxe2-escherichiacoliswineamr) [50]. *Escherichia coli* isolates were assigned to phylogroups using ClermonTyping [51] and sequence types (STs) with PubMLST (https://pubmlst.org/) [44], using the seven-gene Achtman MLST scheme [52].

### Screening for AMR and HM tolerance genetic determinants

All *Escherichia coli* genomes were screened for the presence of AMR genes against the ResFinder database (version 4.4.3) [53] and were grouped into families according to the provided classification. All genomes were further screened for the presence of plasmid replicons using PlasmidFinder (version 2.1.1) [54]. In addition, we used the MetalResistance database from ARGprofiler [55] to identify determinants contributing to HM tolerance. A positive hit was defined for all the software tools when a gene had >80% nt identity over >80 % of the sequence length.

### Assignment of AMR and HM tolerance genes to plasmids

We used a combinational method to estimate the location of the AMR determinants and HM tolerance genes to either a plasmid or chromosomal location in the genome. First, genomes were scanned against a binary plasmid tool plasmidEC [49], which is an ensemble classifier that combines results from Platon [56], RFPlasmid [57] and Centrifuge [58] to classify contigs into those that form part of the chromosome or plasmids. Those contigs putatively identified to form plasmids were used as input for MOB-Suite software [59] using the MOB-Recon option to reconstruct putative plasmid sequences. Briefly, MOB-Suite identifies and assembles plasmid contigs from Mash and blast databases [60]. This combinational method helps overcome some of the limitations of using a single platform to distinguish contigs from the chromosome and plasmids. For example, there is evidence to suggest that using MOB-Suite alone overestimates the presence of AMR genes in the chromosome. Incorporation of plasmidEC specifically helps reduce incorrect attribution of plasmid contigs to the chromosome [61]. Once contigs had been attributed to either the chromosome or a putative plasmid, they were screened against the ResFinder [53] and MetalResistance [55] databases and typed using the PlasmidFinder database [54].

### Long-read sequencing

Hybrid genome assemblies constructed from short- and long-read sequencing data provide confidence when screening large datasets for plasmid-specific sequences. Therefore, a subset of isolates representing all phylogroups (*n*=18) were sequenced using long-read methods. DNA was extracted using the Wizard® HMW DNA Extraction Kit (Promega, WI, USA), following the manufacturer’s instructions and ensuring the DNA was not sheared. DNA concentration was quantified using the QuantiFluor® ONE dsDNA System (Promega). Sequencing libraries were prepared using the Rapid Barcoding Kit version 14, and pooled libraries were sequenced on an Oxford Nanopore Technologies PromethION P2 Solo using an R10.4.1 flow cell. Data acquisition was managed with MinKNOW software, and basecalling was performed using the high-accuracy basecalling model from Dorado using otherwise default parameters. No read length filtering was employed.

A subset of 18 refined genome assemblies (indicated in Table S1) were constructed employing a hybrid approach with the use of Unicycler (version 0.5.0), using default settings [62].

## Results

### *Escherichia coli* isolates from pigs predominantly cluster in phylogroups B1 and A

**Fig. 1.**
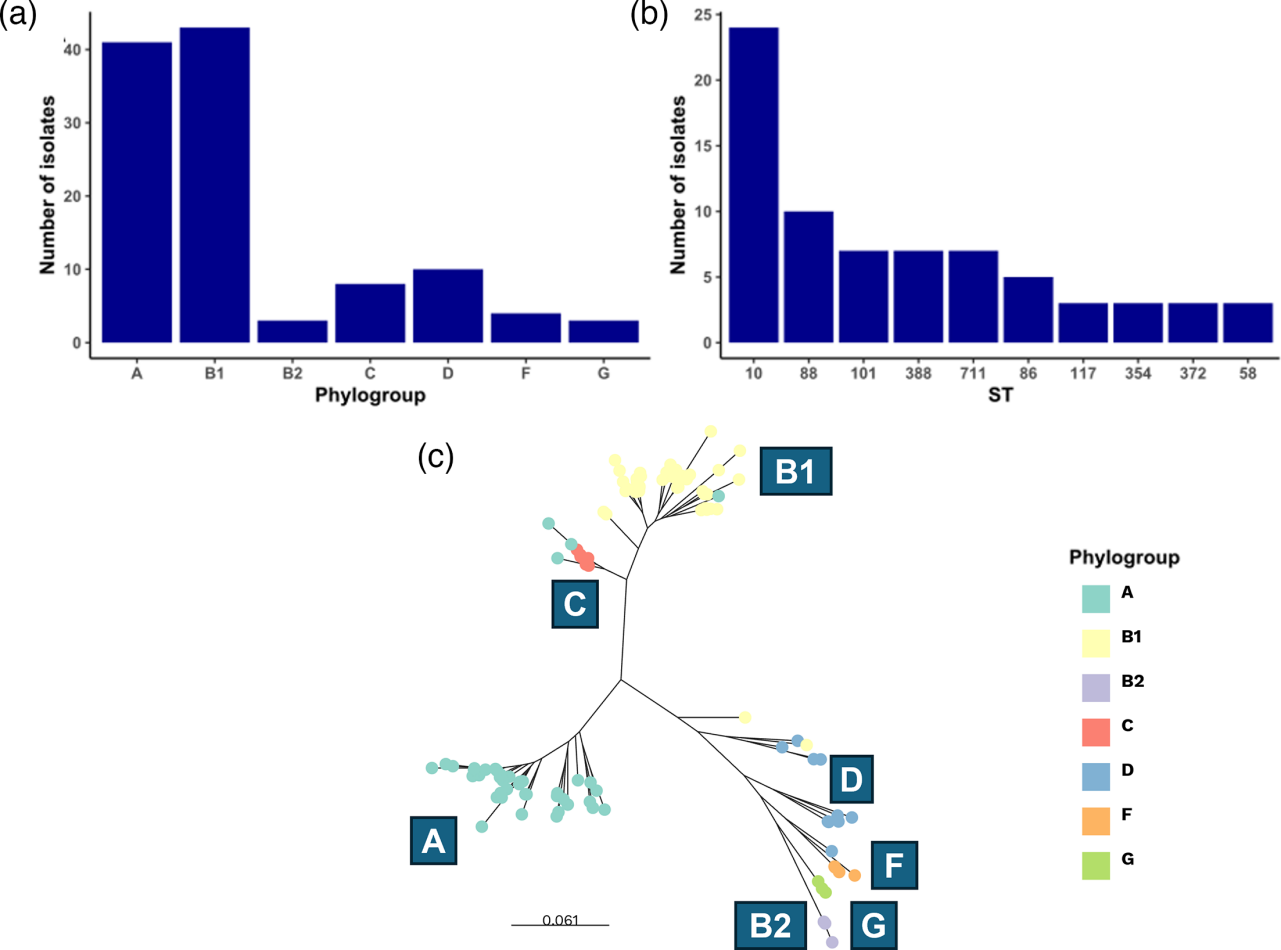
Population structure of *Escherichia coli* porcine strains. (a) Bar plot displaying the isolate number of each phylogroup. (b) Bar plot showing the isolate number of the ten most common STs. (c) Maximum-likelihood phylogenetic tree of 112 *Escherichia coli* strains showing the phylogenetic relationships between the distinct phylogroups. The phylogenetic tree was based on a multiple sequence alignment built from concatenated sequences using the blast algorithm to identify orthologues of genes present in the reference genome *Escherichia coli* K12-MG1655 (*n*=4105 genes), using a gene-by-gene approach. Scale bar is expressed in changes per nt site.

The main objective of this study was to characterize the genomes of 112 porcine *Escherichia coli* isolates from pig farms with a history of diarrhoea. Isolates were principally collected from farms in Catalonia, the leading region in Spain for pig production. AMR determinants and HM tolerance genes were identified and assessed for their potential to pose a public health risk via mobilization and dissemination within the farm environment. There was evidence for variation in the distribution of the pig isolates among six phylogroups. Isolates were assigned to phylogroups with three quarters of *Escherichia coli* isolates in our dataset (75%) distributed among phylogroups B1 (*n*=43; 38.4%) and A (*n*=41; 36.6%) (Fig. 1a, c). Isolates assigned to the remaining phylogroups were less common and included phylogroups D (*n*=10), C (*n*=8), F (*n*=4), B2 (*n*=3) and G (*n*=3). Isolates were also typed according to the Achtman 7-gene MLST scheme and clustered in 33 distinct STs, with ST10 (*n*=24, 21.4%) the most common, followed by ST88 (*n*=10, 8.9%), ST711 (*n*=7, 6.3%), ST388 (*n*=7, 6.3%) and ST101 (*n*=7, 6.3%) (Table S2 and Fig. 1b).

### Genes encoding AMR and HM tolerance determinants are distributed across the population

**Fig. 2.**
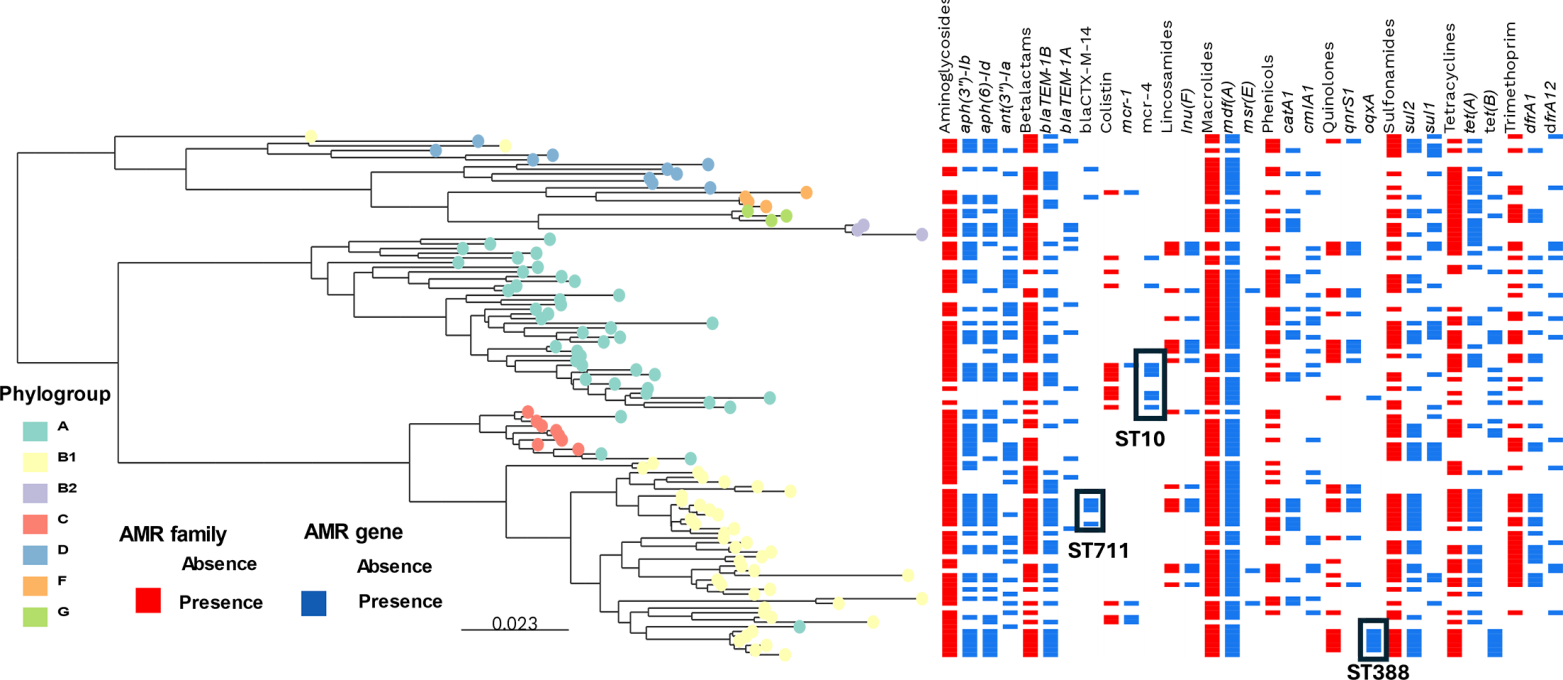
Distribution of AMR genes across population structure. The maximum-likelihood phylogenetic tree of 112 *Escherichia coli* strains was based on a multiple sequence alignment built from concatenated sequences using the blast algorithm to identify orthologues of genes present in the reference genome *Escherichia coli* K12-MG1655 (*n*=4105 genes), using a gene-by-gene approach. The presence/absence of the most common AMR genes grouped by their respective classes against which they confer resistance is mapped against the phylogeny. AMR genes clustered in specific ST clusters are highlighted. Phylogroup was assigned according to the Clermont scheme [50]. AMR determinants were detected according to ResFinder [52]. Scale bar is expressed in changes per nt site.

We compared nt sequences of all 112 porcine *Escherichia coli* genomes against a curated reference database to identify known determinants of AMR. In total, we identified 59 different AMR determinants, and each *Escherichia coli* isolate contained an average of 7.8 AMR genes (range: 1–16), conferring resistance to an average of 5.03 different families of antibiotics (range: 1–9) (Fig. 2 and Table S3), and 92 out of the 112 isolates carried AMR genes from 3 or more distinct resistance classes. We identified AMR determinants from the macrolide (present in 85.7% of the isolates), aminoglycoside (79.4%), beta-lactam (74.1%), sulfonamide (61.6%) and tetracycline (61.6%) families of antibiotics (Table S3). Our most frequently identified AMR determinants were *mdf(A*) (84.8%), *aph(3″)−1b* (52.7%), *bla*_TEM-1b_ (45.6%) and *aph(6)-Id* (45.6%). Notably, we also detected the presence of ESBL genes (*bla*_CTX-M_ type and *bla*_SHV_ type) in 15.2% of isolates, colistin resistance genes (*mcr-1*, *mcr-4* and *mcr-5*) in 13.4% and quinolone resistance genes (*oqxA*, *oqxB*, *qnrB19*, *qnrD1* and *qnrS1*) in 25% of the isolates. Carbapenem resistance such *bla*_OXA-48_ type and tigecycline resistance like *tetX* were not detected. Some AMR determinants were found in clusters of isolates, such as *bla*_CTX-M-14_ or *oqxAB*, which were found exclusively in phylogroup B1 clusters, specifically in isolates from ST711 and ST388, respectively. Six out of eight isolates carrying the *mcr-4* gene clustered in ST10 (phylogroup A) (Fig. 2).

While there have been calls to improve reference databases to better predict the relationship between gene presence and the phenotype [63], using current methods, we identified a high frequency of HM tolerance genes (Table S4), with strains possessing an average of 65.5 genes (range 9–90). We detected at least one HM tolerance gene with activity against a metal used by the agricultural industry in all isolates. For example, every strain contained a gene that is predicted to raise tolerance to either arsenic or cadmium. In addition, putative HM tolerance genes were present in 99% of the isolates against zinc and copper, two metals commonly used in swine production. The least common HM tolerance genes we identified were those conferring resistance to antimony, which were still present in a high number of isolates (91.1%). The high frequency of metal resistance genes we identified suggests that they are part of the core genome of *Escherichia coli*, reflecting their essential roles in metabolism.

### AMR determinants are mostly carried by plasmids, while HM and biocide tolerance genes are chromosomal

**Fig. 3.**
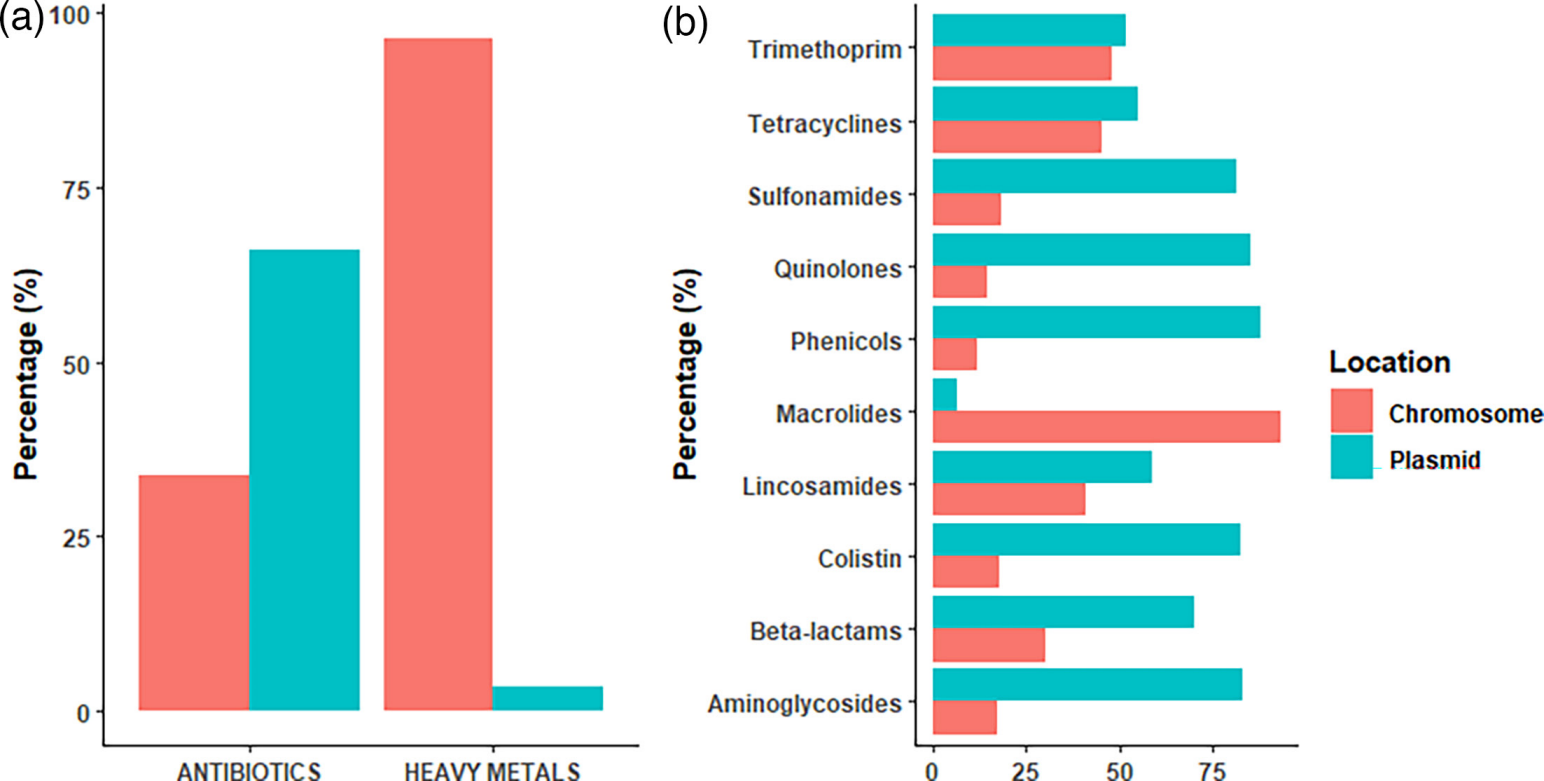
Putative assignment of AMR determinants and HM genes to either the chromosome or plasmids. (a) Bar plot showing the distribution of AMR determinants and HM tolerance genes into chromosomes or plasmids. (b) Bar plot comparing the genome location into chromosome or plasmid for each antibiotic family. Red, chromosome location; blue, plasmid location.

We detected 733 plasmid clusters using a combined bioinformatic approach using MOB-Suite and plasmidEC software that predict plasmid contig sequences in alternative but complementary methods. Where possible, putative plasmids were also typed according to their replicon type (592 of 733 putative plasmids; 80.8%). In total, 193 (26.3%) plasmids included at least 1 AMR resistance determinant. We compared the distribution of AMR determinants and HM genes between plasmid and chromosomal locations (Fig. 3a). AMR determinants were most frequently found on putative plasmids (66.1%), except for the *mdfA* gene (which was exclusively located in the chromosome). Conversely, HM tolerance genes were almost exclusively found in the chromosome (96.4%), except for a minority of genes contributing to tolerance of copper (*pcoA/B/C/D/E/R/S*), silver (*silA/B/C/E/F/P/R/S*), mercury (*merB/C/D/E/P/R/T*) and tellurium (*terW/Z*) found in plasmids. In addition, we observed differences in the distribution of AMR determinants between different antibiotic families (Fig. 3b). While most of the macrolides (95.3%) resistance genes were found in chromosomes, determinants for other antibiotic families were more prevalent in plasmids, particularly those with roles in phenicol (87.9%), quinolone (85.3%), aminoglycoside (82.8%), colistin (82.4%) and sulfonamide (81.5%) resistance.

### Large plasmids tend to carry more AMR genes

**Fig. 4.**
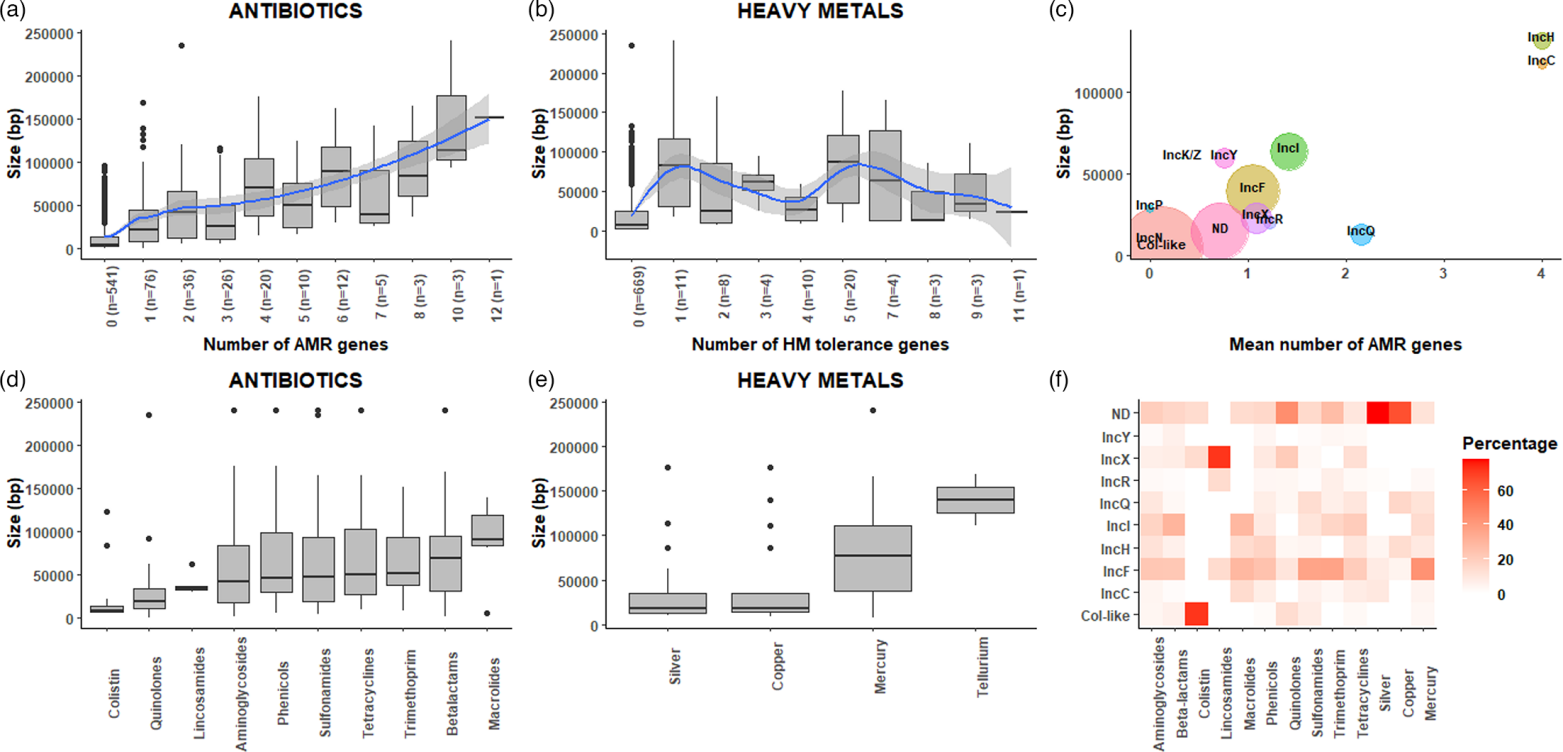
Characteristics of plasmids carrying AMR and HM genes. (a, b) Boxplots showing the distinct plasmid size (bp) according to the number of AMR (a) and HM tolerance genes (b) that contained each plasmid. (c) Size of the plasmids and number of AMR genes contained by each plasmid replicon type. The bigger the circle, the more frequent the plasmid group. (d, e) Boxplots showing the size of the plasmids for each antibiotic (d) and HM (e) family. (f) Heatmap showing the association between the plasmid replicon types and AMR genes grouped into antibiotic/metal families. Only those antibiotic/HM families and plasmids with *n*>5 are visualized.

Plasmid size correlated with the number of AMR determinants (Fig. 4a). However, this relationship was not observed for HM tolerance genes, where most genes were assigned to the chromosome (Fig. 4b). The frequency at which we identified AMR determinants also varied by plasmid replicon type (Fig. 4c and Table S5). Col-like (*n*=295) and IncF (*n*=120) plasmids were the most frequently predicted plasmid types but included a relatively low number of AMR determinants per plasmid (averages of 0.1 and 1.04, respectively). On the other hand, IncH (*n*=12) and IncC (*n*=6) plasmid groups, despite not being very common, contained the greatest number of AMR determinants on average (4).

AMR determinants from certain antibiotic families were accumulated more frequently on larger plasmids; for example, plasmids containing non-chromosomally encoded macrolide or beta-lactam resistance determinants were mostly found on large plasmids, with a median size of 90 986 and 69 550 bp, respectively. AMR determinants conferring putative resistance to lincosamides and quinolones are found on smaller plasmids, with median sizes of 34 441 and 19 303 bp, respectively. Plasmids predicted to carry determinants for colistin resistance are the smallest on average, with a median size of 8631 bp (Fig. 4d). Tellurium and mercury tolerance were present in larger plasmids and copper and mercury genes in smaller ones (Fig. 4e). This reflects a general trend for HM tolerance genes to be found on either small or large plasmids, while AMR determinants were more commonly found on medium-sized plasmids.

Finally, we also investigated the relationship between plasmid types and the AMR gene families (Fig. 4f). As one of the most common replicon types, IncF plasmids were the most common location for most of the AMR gene families. Beta-lactams and non-chromosomally encoded macrolide genes were more commonly found on the larger IncI plasmids, and colistin was highly associated with the smaller Col-like plasmids; lincosamides with IncX plasmids; and quinolones, silver and copper genes with plasmids that could not be typed.

### HM and AMR genes are rarely found on the same plasmid

Despite HM tolerance genes generally being located on the chromosome, mercury, copper, silver and tellurium genes were also found on plasmids. Thus, we sought to determine whether AMR and HM tolerance genes were co-located in the same plasmid sequences. A total of 45 out of 733 plasmids contained both compound types. These plasmids have a higher mean size (84 262 bp) than those which only contain AMR (40 721 bp) or HM genes (21 350 bp). A possible explanation for this is that the most common plasmid replicons were larger in size compared to other less common plasmids, as is the case for IncF (*n*=15), IncH (*n*=7) and IncI (*n*=6) (Fig. S1A). The only exceptions were IncQ plasmids that were relatively common and carried several AMR and HM determinants but are quite small plasmids. The most common resistance families are shown in Fig. S1B. These results reveal that the most common resistance families found in plasmids containing both AMR and HM genes were mercury, despite its lack of use, being represented in 80% of plasmids. This contrasts with the prevalence of more commonly used metals, such as copper, which was present in 15.6% of plasmids.

**Fig. 5.**
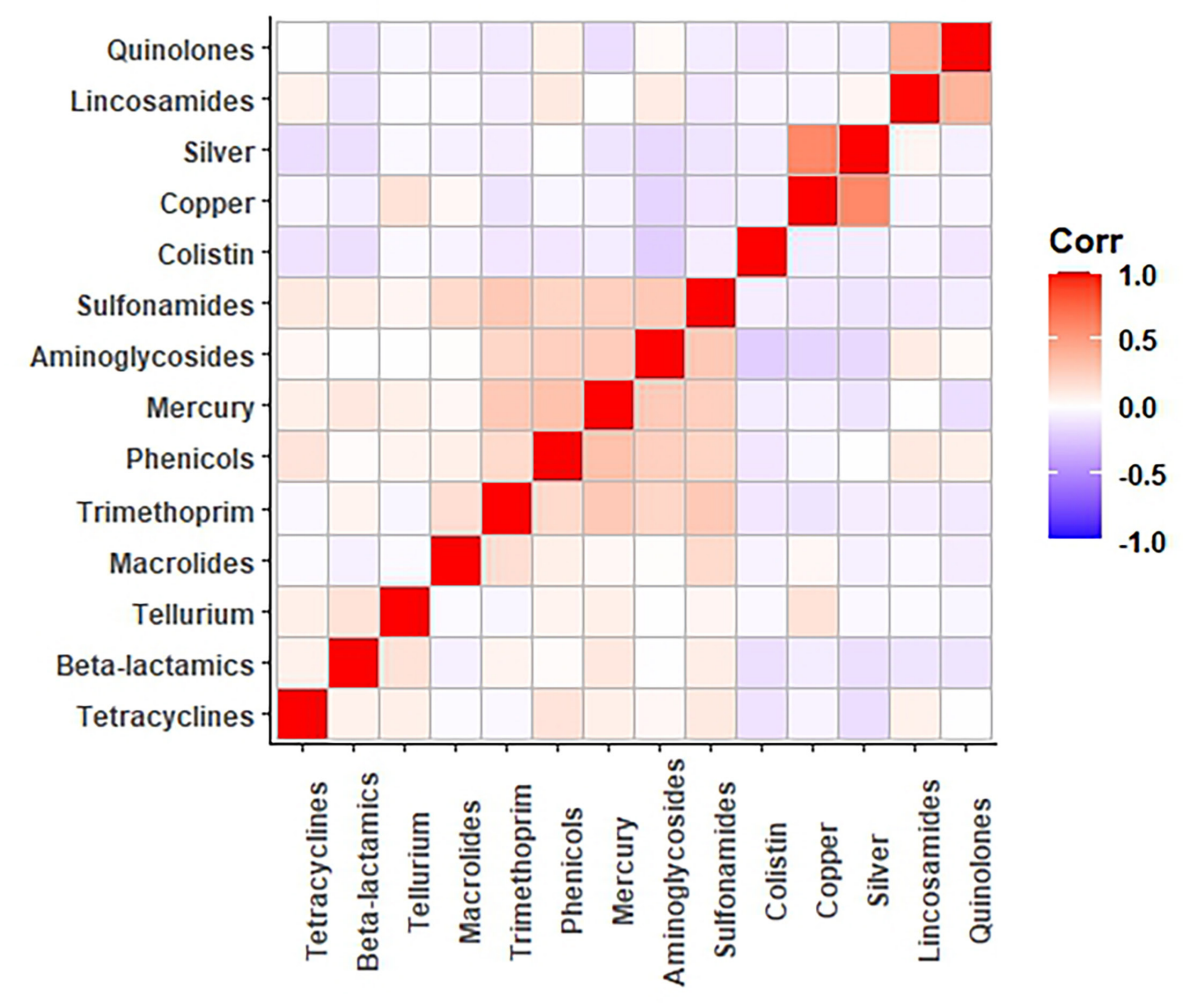
Plasmid co-occurrence matrix showing the co-location of the distinct antibiotic and HM families. Axes are ordered according to hierarchical clustering by group.

We constructed a matrix to identify the co-occurrence of each resistance group on the same plasmid sequences (Fig. 5). The presence of colistin genes was always negatively associated with the presence of any other family, highlighting that these genes tended to be separate from any other resistance groups. Quinolone and lincosamide resistance genes were often discovered on the same plasmids, which tended to occur on IncX-type plasmids (Fig. 4f). With regard to HM tolerance genes, silver and copper genes tended to be located on the same plasmids and negatively correlated with the antibiotic families. Finally, plasmids containing mercury and antibiotic families such as sulfonamides, aminoglycosides, phenicols and trimethoprim are the most frequently found together in the same plasmid, showing the most common route for potential HM–AMR co-selection.

### The *mcr-4* colistin and *qnrD1* quinolone resistance genes are associated with ColE10 and Col3M plasmids, respectively

**Fig. 6.**
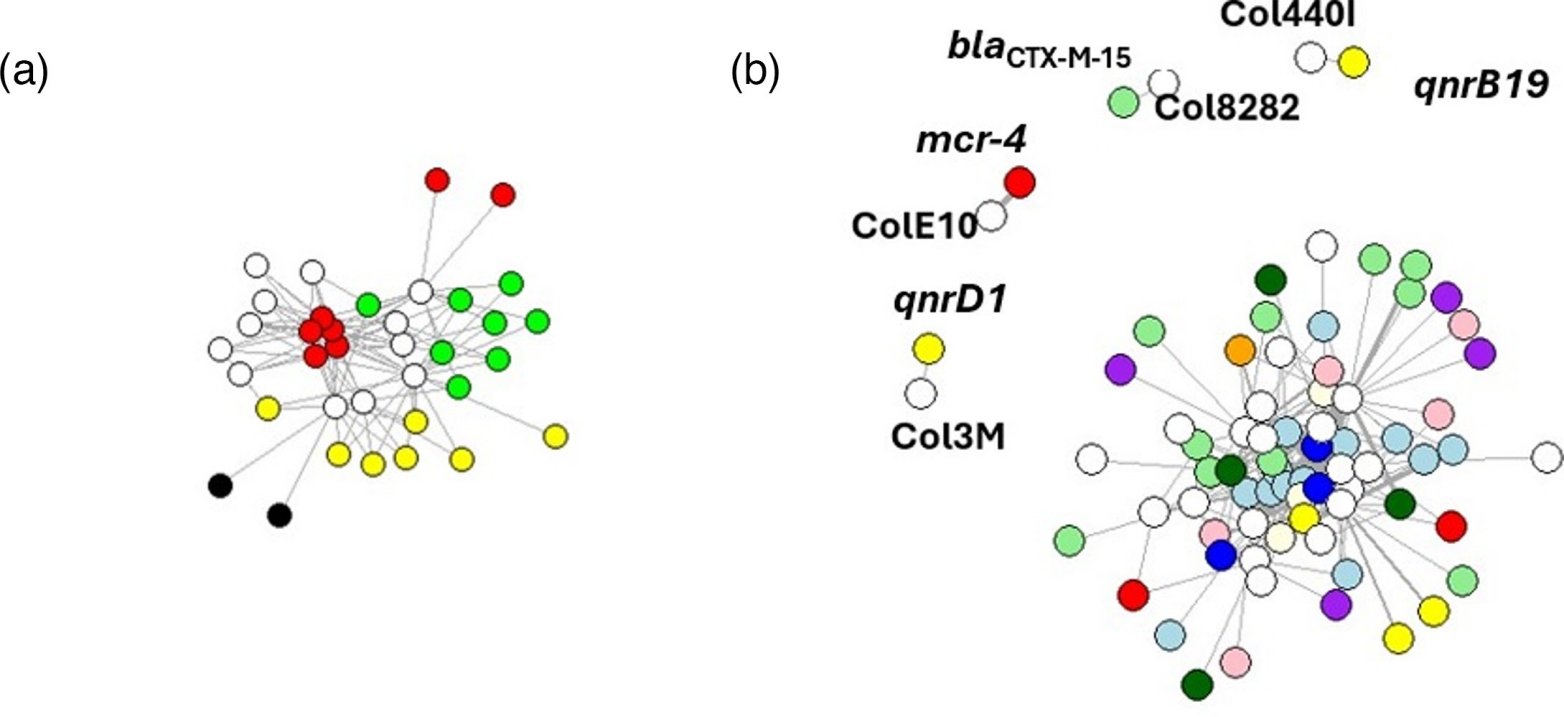
Network analysis of HM tolerance (a) and AMR (b) genes and each location. Genes are connected with the plasmid where they are located. Each circle represented a plasmid (white) or a gene, colour coded by class. (a) HM tolerance genes: red, mercury; green, silver; yellow, copper; and black, tellurium. (b) AMR genes: light blue, aminoglycosides; green, beta-lactams; light yellow, phenicols; pink, trimethoprim; orange, lincosamides; red, colistin; purple, macrolides; yellow, quinolones; dark blue, sulfonamides; and dark green, tetracyclines. Exclusive plasmid–gene associations are highlighted, with their absolute frequencies also indicated. The Fruchterman–Reingold algorithm was used to arrange nodes by balancing repulsive forces between them and attractive forces along edges, producing a clear and evenly spaced network layout, allowing to separately visualize the exclusive plasmid–gene associations.

HM tolerance genes were most typically chromosomally encoded (96.4%), with only a few located on plasmids (3.6%). By clustering genes according to how often they were found on specific plasmids (Fig. 6a), most HM tolerance genes were present uniformly across different plasmid types. Similarly, most AMR genes did not cluster together; however, four genes demonstrated some clustering by plasmid type (Fig. 6b). The quinolone resistance gene *qnrB19* (*n*=1) clustered other genes from the Col440I plasmid, and the ESBL gene *bla*_CTX-M-15_ (*n*=1) was associated with the Col8282 plasmid. These plasmids were exclusively associated with these genes, but, due to the low prevalence, few conclusions can be drawn; more significantly, the quinolone resistance gene *qnr1* (*n*=3) was consistently identified within the Col3M plasmid. The colistin resistance gene *mcr-4* appeared with relatively high frequency (*n*=8) and was consistently found in the ColE10 plasmid. Notably, these plasmids did not carry any other AMR or HM tolerance genes.

### Validation of plasmid identification through long-read sequencing

**Fig. 7.**
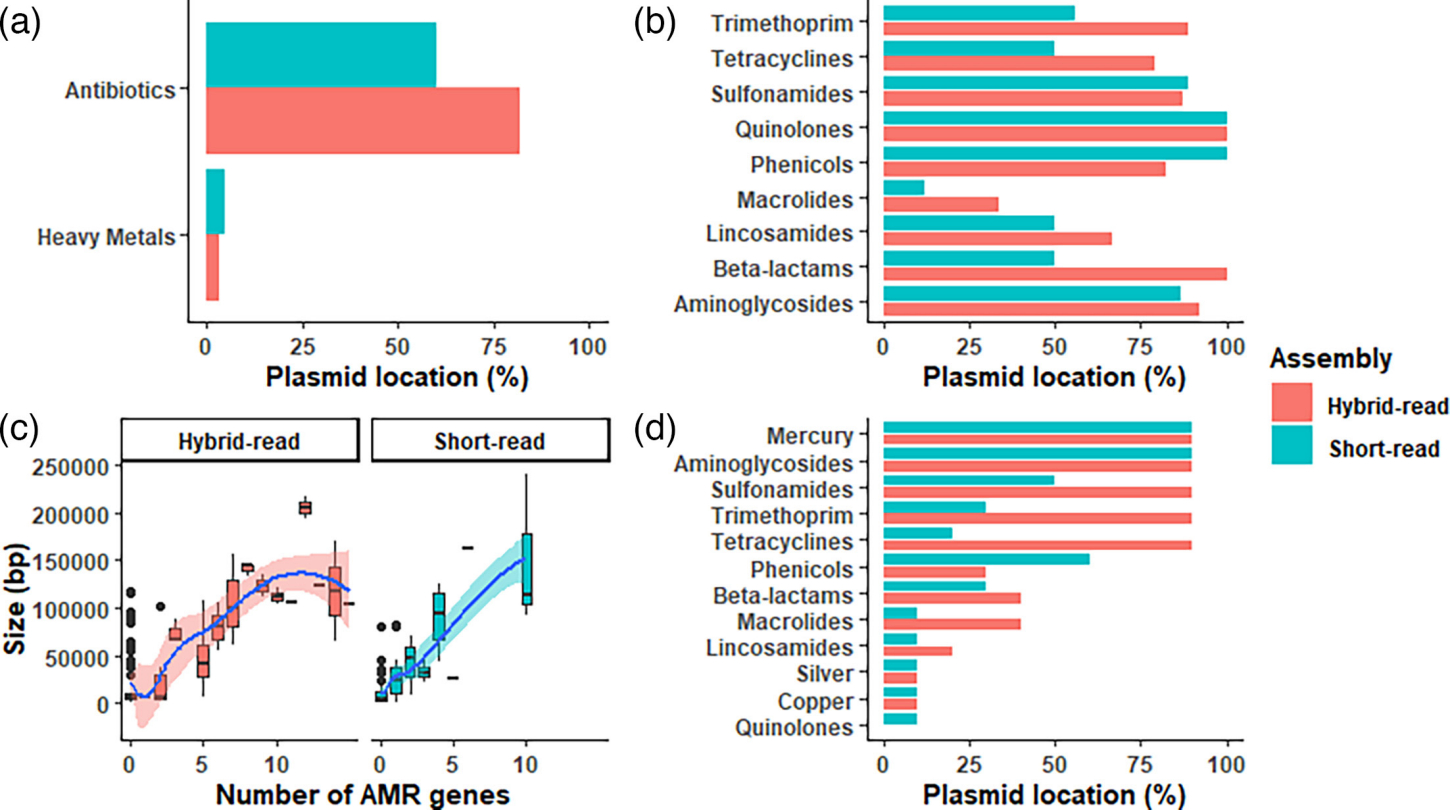
Result validation using a comparison of a subset of 18 isolates between hybrid and short-read assemblies. (a, b) Bar plots comparing the location in plasmids for AMR and HM tolerance genes (a) and specifically for each antibiotic family (b). (c) Boxplots showing the distinct plasmid size (bp) according to the number of AMR genes that each plasmid contained. (d) Bar plot showing the most common antibiotic/HM families in plasmids containing both compounds.

To support our combinational approach to plasmid identification, we sequenced a subset of isolates representing all phylogroups with long-read sequencing methods, constructed hybrid assemblies (*n*=18) and compared them with short-read assemblies. This approach also placed most AMR determinants in plasmids (even in a higher proportion), while HM tolerance genes were chromosomally encoded (Fig. 7a). Antibiotic families follow a similar pattern to the short reads, even though the location in plasmids was again higher for most of the classes (Fig. 7b). Moreover, plasmid length positively correlated with the number of AMR genes present (Fig. 7c), and the possible co-selection of HM and AMR genes was mainly mediated by an environmental polluter not used in swine production such as mercury (Fig. 7d), validating the main findings with the hybrid assemblies.

## Discussion

As one of the earliest identified microorganisms, *Escherichia coli* is the most well-studied and characterized bacteria [64]. With a broad host range and widespread distribution, *Escherichia coli* has often been used as a sentinel species to study bacterial diversity [32,65], the effects of horizontal gene transfer [66,67] and the dissemination of AMR [3,68]. In this study, we sequenced and screened 112 *Escherichia coli* isolates from a region in Spain with one of the highest densities of pig farms. Previously, studies identified phylogroup A as the most common in pigs, followed by B1 [69,71]. Our results are consistent with this, with other phylogroups represented by less than 10% of isolates. Several plasmids were identified in each strain (average: 6.5), which is higher than observed in many other studies of bacterial species, such as *Salmonella* [72], but in consonance with other studies of livestock *Escherichia coli* genomes [73,74].

We identified multiple AMR determinants per isolate, conferring predicted resistance to an average of 4.5 antibiotic classes. MDR isolates were widespread in our collection (82.1%), conferring resistance to three or more antibiotic families [75]. Of particular public health concern, genes that can confer resistance to last-resort antibiotics used in human medicine were found in multiple isolates. For instance, ESBL genes were present in 17% of our isolates, including *bla*_CTX-M-14_, which is commonly reported in swine [36,76,79] and was located mainly in the ST711 clone, found in different sources such as human clinical samples [80] or horses [81]. In contrast, *bla*_CTX-M-1_, which is prevalent in Europe [82], was absent. Additionally, low numbers of ESBL genes related to human clinical isolates were detected, such as *bla*_CTX-M-15_ and *bla*_CTX-M-27_, associated with pandemic lineage ST131 in hospital settings [83,84].

Resistance determinants with activity to last-resort antibiotics, such as carbapenem or tigecycline, which have previously been reported in pigs from different countries, mainly in Asia, such as *bla*_OXA-48_ [85], *bla*_NDM_ [86,87] or *tetX* [88], were not detected. Given that carbapenems are not licensed for use in food-producing animals in the European Union and other parts of the world, the absence of carbapenem-resistant genes supports the continued restriction of their use in veterinary medicine to prevent the emergence and spread of resistant strains through the food production chain. Colistin resistance genes (*mcr-1*, *mcr-4* and *mcr-5*) were found in 13% of isolates, with *mcr-4* being the most frequent, predominantly in ST10 clones, aligning with other studies conducted in southern Europe, including Spain [36], Portugal [89] and Italy [90]. Given that *mcr-1* is the most frequently occurring in pigs globally [13], the results presented here suggest that the frequency of *mcr* genes may vary depending on the geographical region studied.

Plasmids were the primary vehicle for the transmission and dissemination of AMR genes [91]. This mirrors findings in avian pathogenic *Escherichia coli*, where isolates primarily acquire virulence genes through the acquisition of plasmids [92]. However, the carriage of HM tolerance genes on plasmids was infrequently observed in our collection. This likely reflects the essential role of metal ions in core metabolism. For example, many enzymes require metal ions as co-factors, such as RNA polymerase that requires magnesium ions for active function. However, high concentrations of HM ions are toxic to the cell, and metal ion homeostasis is therefore carefully regulated. Bacteria possess a suite of genes to aid metal ion uptake, storage and efflux, all under the tight control of regulators [93,94].

Some studies have found a correlation between HM use/exposure (especially zinc) and AMR levels in pig faeces [95,97], and it has been suggested that these genes are carried together on the same mobile genetic elements [98]. However, we observed limited co-carriage of AMR determinants and HM tolerance genes on the same plasmid. Another hypothesis is that metal exposure alters the swine microbiota [99,101], selecting for isolates with chromosomally encoded AMR genes [102,103]. Alternatively, oxidative stress might increase AMR gene expression and conjugation [104], with copper specifically raising the minimum inhibitory concentration in *Escherichia coli* [105]. Further research is needed to explore these hypotheses and understand how metals exacerbate problems associated with AMR. Nevertheless, co-selection, although rare, was observed, with mercury – an environmental polluter not used in swine production – being the most implicated metal, as previously demonstrated in swine [106] and human isolates [107,108]. This suggests an environmental origin for these plasmids, underscoring the importance of the One Health approach.

Transmission of AMR *Enterobacteriaceae* between human and livestock, at least in developed countries, is unclear [17,109], although plasmid sharing is possible [110,111]. We analysed plasmid epidemiology by replicon type and size, finding IncF and Col-like plasmids to be the most common, consistent with previous studies [3,36, 71]. Only 26.3% of plasmids contained AMR genes, indicating that most plasmids are not involved in AMR transmission. Generally, plasmid size correlated with the number of AMR genes [112,113], with colistin resistance genes typically found on smaller Col-like plasmids, often without other genes. These results contradict the findings in China [114], where most studies have focused on the *mcr-1* gene. The most common *mcr* gene in our dataset was *mcr-4*, suggesting a difference in the epidemiology between the *mcr-1* genes and the frequency of co-selection with other AMR determinants. Sulfonamide, aminoglycoside, phenicol, trimethoprim and mercury resistance genes tended to co-occur on the same plasmid, which is consistent with other class 1 integrons [69,70], and pose a public health concern since these classes are commonly used to treat porcine bacterial infections.

We also studied the genetic context of AMR genes on specific plasmids. For specific examples such as colistin, stopping antibiotic use might not be enough to eliminate resistance genes if they are co-located with other beneficial genes [115]. Targeting specific plasmid types could help reduce AMR transmission [116]. Findings from our study support previous work reporting *qnrD* on Col3M plasmids [117] and *mcr-4* as the exclusive resistance gene on ColE10 plasmids [36]. While the *mcr-4* gene has not been widely reported, this gene appears to be relatively frequent in the Iberian Peninsula [36,89, 118], and consequently, targeting ColE10 plasmids could represent an alternative way to control the dissemination of colistin resistance in the farm environment. Identification of *mcr-4* genes in samples from wildlife in other areas of Catalonia [119] suggests environmental contamination and the need for improved control strategies.

There were limitations to our study. Despite the efficacy of MOB-Suite [59] to reconstruct plasmid sequences from short-read data [22,72, 120], ambiguous contigs are assigned to the chromosome – which would underestimate the number of AMR genes found on plasmids in our collection. This was partially overcome by using a combinatorial approach with supporting data from plasmidEC. Although challenges remain in reliably identifying plasmids from genomic data using short reads alone, we also verified our assignment of contigs using long-read methods, which broadly agreed with our categorizations [121]. Samples were exclusively obtained from Catalonia, enabling a precise characterization of the local situation; however, results may differ in other regions. Our findings could also be influenced by sampling bias, as the isolates were randomly selected from previous studies. Ideally, a stratified sampling approach covering diverse locations within the geographic area would have been more appropriate. Finally, the findings of this study should not be only related to pig farms, but rather framed within the One Health concept. High levels of AMR have been found in Spain not only in human clinical samples but also in food samples [122], companion animals [123] or wildlife [124], which reinforce the idea of the need for multidisciplinary surveillance.

In summary, this study genomically characterizes 112 *Escherichia coli* isolates from a high-density pig population in Catalonia. We found a high frequency of AMR genes, including those conferring resistance to critical antibiotics. Plasmid size correlated with AMR gene quantity, and colistin resistance genes were often alone on small plasmids. HM tolerance genes were common but rarely co-carried with AMR genes on the same plasmid. Further research on oxidative stress and other hypotheses is needed to understand how metals contribute to AMR. Finally, specific plasmids, such as ColE10 carrying *mcr-4*, may provide new targets for controlling AMR in this region.

## supplementary material

10.1099/mgen.0.001371 Uncited Supplementary Material 2.

10.1099/mgen.0.001371 Uncited Fig. S1.

## Abbreviations

AMR: antimicrobial resistance
CIA: critically important antimicrobial
ESBL: extended-spectrum *β*-lactamase
HM: heavy metal
MDR: multidrug resistant
ST: sequence type
