# Supplementary material for: Characterization of antibiotic determinants and heavy metal resistance genes in Escherichia coli from pigs in Catalonia
Source: Microb Genom. 2025 Mar 25;11(3):001371. doi: 10.1099/mgen.0.001371 (PMC11937225; doi:10.1099/mgen.0.001371)
Supplement: Uncited Fig. S1. [file mgen-11-01371-s002.pdf]

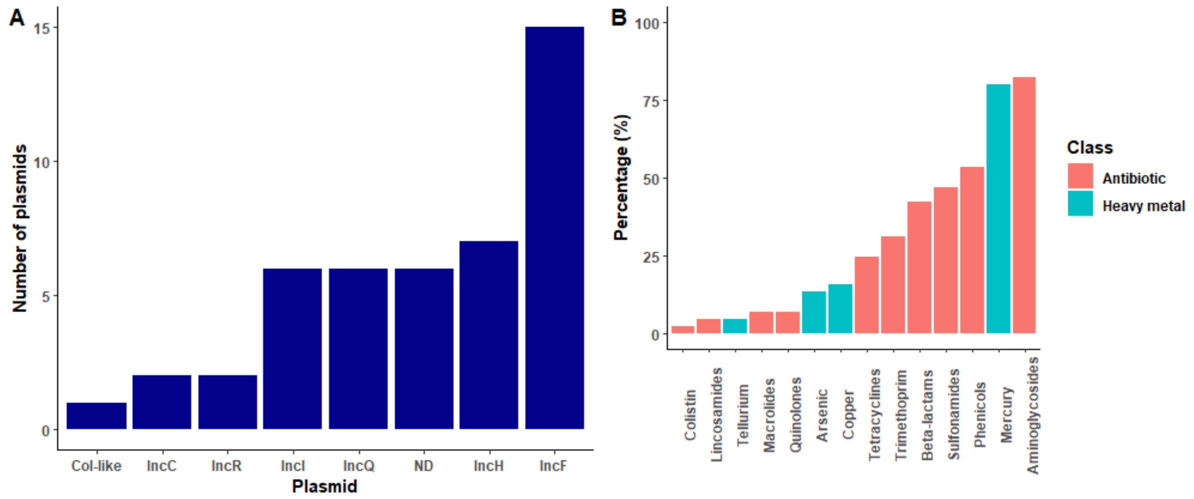

Figure S1: Characteristics of plasmids sharing AMR and HM genes. A: Barplots showing the most common plasmid replicon types (A) and the most common families (B) in those plasmids containing AMR and HM genes. Red: antibiotic classes. Blue: Heavy metals.
